# Common-Ion Effect Triggered Highly Sustained Seawater Electrolysis with Additional NaCl Production

**DOI:** 10.34133/2020/2872141

**Published:** 2020-09-24

**Authors:** Pengsong Li, Shiyuan Wang, Imran Ahmed Samo, Xingheng Zhang, Zhaolei Wang, Cheng Wang, Yang Li, Yiyun Du, Yang Zhong, Congtian Cheng, Wenwen Xu, Xijun Liu, Yun Kuang, Zhiyi Lu, Xiaoming Sun

**Affiliations:** ^1^State Key Laboratory of Chemical Resource Engineering, Beijing Advanced Innovation Centre for Soft Matter Science and Engineering, College of Chemistry, Beijing University of Chemical Technology, Beijing 100029, China; ^2^Chinese Research Academy of Environmental Sciences Institution, Beijing 100012, China; ^3^State Nuclear Electric Power Planning Design & Research Institute Co., Ltd., Beijing, China; ^4^Ningbo Institute of Materials Technology and Engineering, Chinese Academy of Sciences, Ningbo, 315201 Zhejiang, China; ^5^Center for Electron Microscopy and Tianjin Key Lab of Advanced Functional Porous Materials, Institute for New Energy Materials & Low-Carbon Technologies, School of Materials and Engineering, Tianjin University of Technology, Tianjin 300384, China; ^6^University of Chinese Academy of Sciences, Beijing 100049, China

## Abstract

Developing efficient seawater-electrolysis system for mass production of hydrogen is highly desirable due to the abundance of seawater. However, continuous electrolysis with seawater feeding boosts the concentration of sodium chloride in the electrolyzer, leading to severe electrode corrosion and chlorine evolution. Herein, the common-ion effect was utilized into the electrolyzer to depress the solubility of NaCl. Specifically, utilization of 6 M NaOH halved the solubility of NaCl in the electrolyte, affording efficient, durable, and sustained seawater electrolysis in NaCl-saturated electrolytes with triple production of H_2_, O_2_, and crystalline NaCl. Ternary NiCoFe phosphide was employed as a bifunctional anode and cathode in simulative and Ca/Mg-free seawater-electrolysis systems, which could stably work under 500 mA/cm^2^ for over 100 h. We attribute the high stability to the increased Na^+^ concentration, which reduces the concentration of dissolved Cl^−^ in the electrolyte according to the common-ion effect, resulting in crystallization of NaCl, eliminated anode corrosion, and chlorine oxidation during continuous supplementation of Ca/Mg-free seawater to the electrolysis system.

## 1. Introduction

Hydrogen, with a high gravimetric energy density of 142 MJ/kg, is considered one of the most promising clean energy carriers [1–3]. Electrochemical water splitting is a promising and green pathway that converts the sustainable-renewable energy resources into H_2_ [4–7]. Recently, seawater splitting [8–10] receives great attention due to the abundant resources (~97% of water in our planet) on earth and avoiding the competition to drinking water [11–13], as well as concentrating high-value elements to maximize the utilization of seawater. Seawater exhibits an average salinity of ~3.5% (~0.599 M), in which NaCl is the predominant species. As a consequence, seawater splitting encounters challenges such as undesirable chlorine oxidation reaction [14] and severe corrosion of electrodes [8, 15]. In contrast to the tremendous efforts devoted recently to develop highly active electrocatalysts (e.g., metal, metal phosphide [16], doped nanocarbons [17], and Ni-Fe-C [18] for hydrogen evolution and metal hydroxide [19], carbonate [14], and nitride [10] for oxygen evolution), few works focused on the electrolyte design so far. Indeed, contemporary works employed mimic seawater in alkaline electrolyte (e.g., 0.5 M NaCl +1 M KOH) as water source and reported attractive electrolysis performance [9, 10, 20]. However, pure water instead of seawater is usually fed in long-term stability testing (>100 hours) for preserving the ion concentration in the electrolyte, which is not practical.

**Figure 1:**
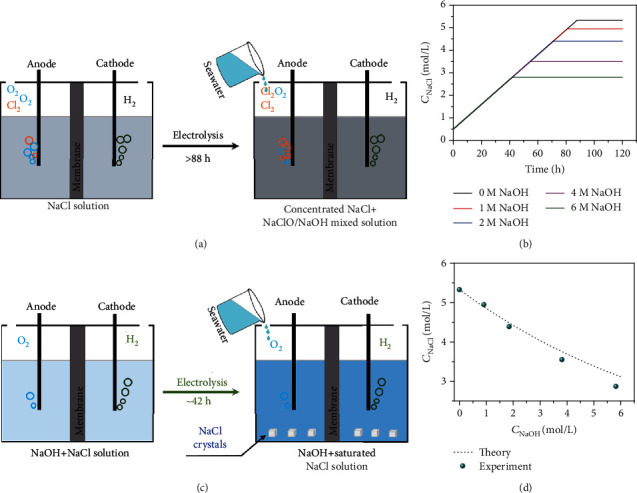
Highly sustained seawater electrolysis using the common-ion effect. (a) The schematic diagram depicts the challenge of neutral seawater electrolysis. The electrolysis time comes from (b). (b) The concentration changes of NaCl during the seawater electrolysis with different initial concentrations of NaOH. Calculation conditions: the electrode area is about 0.2 m^2^; the volume of electrolyte is 3 L; the current density is 500 mA/cm^2^. (c) The common-ion effect is introduced into seawater electrolysis to improve the electrochemical performance and produce triple product (H_2_, O_2_, and NaCl). (d) The solubility of NaCl with different NaOH concentrations.

Direct electrolysis of real seawater under neutral condition is a promising way [21] but would face severe chlorine evolution reaction on the anode. During continuous neutral seawater electrolysis (Figure 1(a)), Cl^−^ would accumulate gradually, which accelerates the electrode corrosion and favors undesirable chloride oxidation (ClOR) to chlorine/hypochlorite. Even worse, long-term electrolysis of seawater in this case would cause saturation of NaCl in ~88 hours (Figure 1(b) and S1, initiate and feed with Ca/Mg-free seawater, electrode area about 0.2 m^2^, 3 L of electrolyte, *I* = 500 mA/cm^2^). The final concentration of NaCl would be ~5.3 M, which would cause severe electrode corrosion [9].

Compared to the neutral system, seawater electrolysis in alkaline environment can possibly alleviate the above problems, as excessive OH^−^ around the anode would thermodynamically favor OER and simultaneously suppress ClOR, according to the Nernst equation [22]. However, this system would also accumulate Cl^−^ to oversaturation in long-term electrolysis. Therefore, an ideal electrolyte should simultaneously have high [OH^−^] and low [Cl^−^] (even saturated) [20], which is the key for sustainable and stable seawater electrolysis system, but remains a big challenge.

Keep this in mind, “common-ion effect” is introduced to the electrolyte design. It is basically an equilibrium used to describe the solubility of a salt in the presence of another salt with a common ion. That is, the product of the maximum concentrations of two dissociated ions (one positive and one negative) is a constant. Larger product would result in oversaturation and nucleation of the salt. Increasing the concentration of one ion would decrease the max solubility of the other ion. Thus, in seawater electrolysis system, a clear consequence is that adding NaOH (Na^+^) can reduce the saturated concentration of NaCl (Cl^−^). This means OER process could be favored while ClOR and anode corrosion could be depressed. The common-ion effect can thus keep the Cl^−^ concentration at a low level during the long-term seawater electrolysis with continuous feeding of Ca/Mg-free seawater, meanwhile shortening the time to oversaturation (Figure 1(b), the concentration of NaCl as a dependence of NaOH concentration from 1 M to 6 M) and eliminating anode corrosion and chlorine evolution by crystallization of NaCl, making the triple production (e.g., H_2_, O_2_, and NaCl) feasible (Figure 1(c)). As estimated following the above way, the electrolysis time for NaCl to reach saturation is roughly halved (~42 hours) when adding 6 M NaOH to the Ca/Mg-free seawater electrolyte.

Herein, we used 6 M NaOH as electrolyte for seawater splitting system (higher NaOH concentration would decrease the conductivity of the electrolyte) to depress the solubility of NaCl. 0.5 M NaCl solution was used as mimic seawater, or Ca/Mg-free (pretreated) seawater was used as feeding solution. With a highly active, corrosion-resistive, bifunctional NiCoFeP material as both anode and cathode, the electrolyzer could work selectively and stably for H_2_ and O_2_ production in NaCl saturation conditions, as well as crystallization of NaCl during continuous addition of Ca/Mg-free seawater. The reduced Cl^−^ concentration could effectively alleviate the corrosion problem of the anode and depress ClOR, and thereby, the system can work stably under a high current density of 500 mA/cm^2^ for 100 h with only 0.06 V cell voltage increase. Furthermore, the NaCl crystal production rate can be increased by six times in the catholyte when a Nafion membrane was applied, as the Nafion membrane only transfers Na^+^ across the membrane from anode to cathode. This work demonstrates a practical electrolyte design for future practical seawater electrolysis.

## 2. Results and Discussion

The common-ion effect is demonstrated in Figure S2: when NaOH were added into the saturated NaCl solution (~5.3 M), the NaCl crystals precipitated from the solution due to the increased concentration of Na^+^. We collected the NaCl crystals by filtration and listed the weights in Table S1. The relationship between the solubility of NaCl and the concentration of NaOH is plotted in Figure 1(d). The solubility of NaCl (i.e., Cl^−^ concentration) significantly decreased from ~5.3 M to ~2.8 M after the addition of 6 M NaOH due to the common-ion effect (Figure 1(d)). In order to highlight the importance of the “common-ion effect,” we conduct a control experiment by adding KOH in saturated NaCl. When KOH powder was added to the saturated NaCl solution, most of the formative crystals were KCl (Figure S3), suggesting that K^+^ would not affect the “common-ion effect” induced by Na^+^.

**Figure 2:**
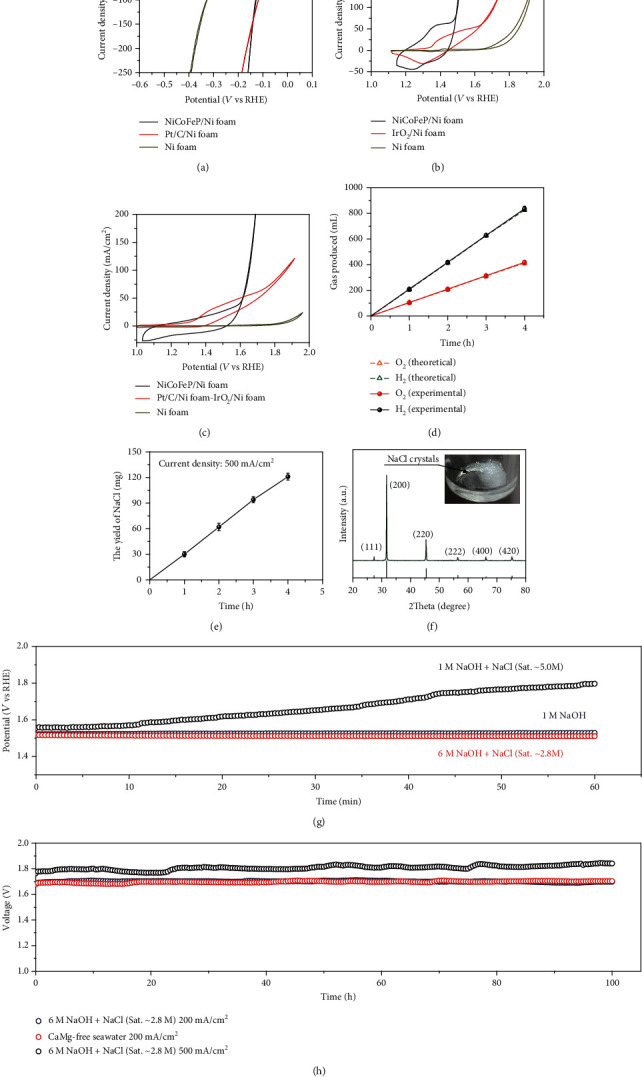
Electrochemical performance of the NiCoFeP electrode. (a–c) CV curves of NiCoFeP and commercial catalysts for HER, OER, and overall water splitting after *iR* compensation. (d) The generated amounts of H_2_ and O_2_ from water electrolysis using NiCoFeP array as the cathode and anode electrodes at different time intervals with the current density of 500 mA/cm^2^ in 6 M NaOH + NaCl (Sat. ~2.8 M) electrolyte and corresponding yield of NaCl crystals (e) during the water electrolysis. Error bars represent standard deviations from multiple measurements. (f) XRD pattern of the product formed (NaCl) in the electrolyte after electrolysis, and the inset in the digital image depicts the NaCl crystals at the bottom of the electrolyzer. (g) Chronopotentiometry curve of OER (NiCoFeP) for 60 minutes at the current density of 200 mA/cm^2^ using different electrolytes with *iR* compensation, indicating that the stability can be improved by suppressed Cl^−^ concentration. (h) Chronopotentiometry curve of overall water splitting (NiCoFeP) for 100 h at different current densities with *iR* compensation using 6 M NaOH + NaCl (Sat. ~2.8 M) and Ca/Mg-free seawater (real seawater pretreated by NaOH and Na_2_CO_3_) as the electrolyte.

Electrochemical performance were evaluated in 6 M NaOH with saturated NaCl (6 M NaOH + NaCl (Sat. ~2.8 M)) electrolyte. The ternary NiCoFe phosphide (NiCoFeP) nanoarrays grown on the Ni foam was selected as both the cathode and the anode. The electrodes were prepared by hydrothermal synthesis of NiCoFe hydroxides followed by phosphating (please see the experimental procedures for detail). The structure evolution from hydroxide to phosphide was evidenced by X-ray diffraction (XRD, Figure S4a). After the phosphorization process of NiCoFe hydroxide array, the peaks belonging to NiCoFe hydroxide disappeared in XRD pattern (Figure S4a), while two new peaks appeared which could be indexed to Ni_2_P or Co_2_P [23, 24] belonging to a hexagonal crystalline structure. No other phase was detected, demonstrating the complete transformation from hydroxide to phosphide. The energy dispersive X-ray spectroscopy (EDX) elemental mapping of one piece of NiCoFeP (Figure S4c-S4d) proved that Ni, Co, Fe, and P elements were uniformly distributed, and electron diffraction (ED) pattern (Figure S4b) showed single crystalline structure, suggesting that NiCoFeP was in a single crystalline phase (hexagonal). In addition, the ICP test indicated that the percentages of Ni, Co, and Fe among the metallic elements were 25.9 at%, 55.6 at%, and 18.5 at%, respectively, suggesting a cobalt-rich NiCoFe phosphide composition. Cyclic voltammetry (CV) was recorded in a standard three-electrode system. The performance is shown in Figure S5 and Figure 2. For HER, the NiCoFeP array exhibits an overpotential of -129 mV at the current density of -100 mA/cm^2^ (Figure 2(a)), which was far better than that (316 mV) of Ni foam and closer to that (119 mV) of Pt/C, indicating that the NiCoFeP affords significantly high HER activity. The NiCoFeP array also exhibited a remarkable OER activity (Figure 2(b)), with a lower onset potential than the commercial IrO_2_ and Ni foam. CV curves revealed that a small overpotential (~272 mV) for achieving a high current density (100 mA/cm^2^) was needed on a NiCoFeP array electrode. These indicate that NiCoFeP material can be used as a bifunctional catalyst for overall water splitting. The overall water splitting test was performed in a two-electrode cell assembling with NiCoFeP as both the cathode and the anode. The CV curves of the overall water splitting (Figure 2(c)) indicated a cell voltage of 1.64 V at the current density of 100 mA/cm^2^ in the 6 M NaOH + NaCl (Sat. ~2.8 M) electrolyte. It is notable that the bifunctional NiCoFeP array electrode showed superior performance over the couple of commercial Pt/C and IrO_2_.

Further, the three products of electrolysis were also examined. The gas products were collected by the drainage method (measure the volume). When collecting O_2_, the gas would pass through another strong base solution to absorb the Cl_2_ that might be produced in the anode.  Figure 2(d) suggests that the Faradaic efficiencies were near 100% for both HER and OER possessing the average volume ratio of H_2_ to O_2_ which is 2.01. It eliminated chloride oxidation, which was essential to achieve efficient seawater splitting [14]. Different from previous reports, the crystallization of NaCl was observed and collected (Figures 2(e) and 2(f)). The XRD pattern confirms the formation of phase pure NaCl (PDF no. 75-0306). Extension of electrolysis time would yield more NaCl crystals due to consumption of water.

Indeed, the high alkalinity (e.g., 6 M NaOH) of electrolyte is critical for building a robust seawater electrolysis system. Two different electrolytes, 1 M NaOH and 1 M NaOH + NaCl (Sat. ~5.0 M), were evaluated using the same catalytic electrode (NiCoFeP array) to experimentally demonstrate the electrolyte advantage, as shown in Figure 2(g). It is demonstrated that the seawater electrolysis system worked stably in both 6 M NaOH + NaCl (Sat. ~2.8 M) and 1 M NaOH electrolytes, while the potential would rise very quickly in the 1 M NaOH + NaCl (Sat. ~5.0 M) electrolyte. The gas produced by the anode was collected every 20 minutes to calculate the Faradaic efficiency (FE) of O_2_ during the stability test. In the 1 M NaOH electrolyte and 6 M NaOH + NaCl (Sat. ~2.8 M) electrolyte, the FE of O_2_ could maintain at about 100% during the 60-minute test, which meant that there was no chloride oxidation with such high concentration of OH^−^. When the electrolyte was changed to1 M NaOH + NaCl (Sat. ~5.0 M), the FE of O_2_ was 92% in the first 60 min. Even worse, the FE decreased from 92% to 46-65% by prolonging electrolysis time (Figure S6), indicating that a considerable portion of current density (~100 mA/cm^2^) was used to corrode electrode assisted with Cl^−^. The above stability test and FE results clearly illustrated that, under the condition of saturated Cl^−^, the electrodes were resistant to corrosion only at a high concentration of NaOH. This is because the concentration of Cl^−^ can be suppressed by increasing the concentration of NaOH due to the common-ion effect in our electrolyte system.

It is found that, in our system, the electrolysis current density can be further increased without affecting the stability. As shown in Figure 2(h) and S5, the chronopotentiometry curve showed negligible cell voltage attenuation (<0.06 V) under the constant current density of 200 mA/cm^2^ and 500 mA/cm^2^ after 100 h electrolysis. To push our design closer to the practical application, the real seawater was used as the single water source for electrolysis. To remove Ca^2+^ and Mg^2+^, the real seawater was pretreated by adding NaOH and Na_2_CO_3_ to 6 M and 0.5 M, respectively. Afterwards, the Ca/Mg-free seawater was used as the electrolyte after simple filtration. The Ca/Mg-free seawater electrolysis system exhibited similar performance to that in 6 M NaOH + NaCl (Sat. ~2.8 M), as shown in Figures 2(h) and S7. The above results demonstrated that the presence of Cl^−^ in the 1 M NaOH solution would cause a certain activity attenuation of catalysts (Figure S8a), while high alkalinity (6 M NaOH) could avoid this decay (Figure S8b). The NiCoFeP electrode was also selected as the anode and cathode for the stability test in 6 M NaOH + NaCl (sat. ~2.8 M) electrolyte with continuous feeding of Ca/Mg-free seawater every 12 h. As shown in Figure S9a, the chronopotentiometry curve showed negligible cell voltage attenuation under the constant current density of 20 mA/cm^2^ after 112 h electrolysis. The OER and HER CV curves of NiCoFeP electrode before and after chronopotentiometry test are shown in Figures S9b and S9c. There was no obvious performance degradation for NiCoFeP in OER and HER, demonstrating the excellent stability of the NiCoFeP electrodes in long-term overall splitting chronopotentiometry.

**Figure 3:**
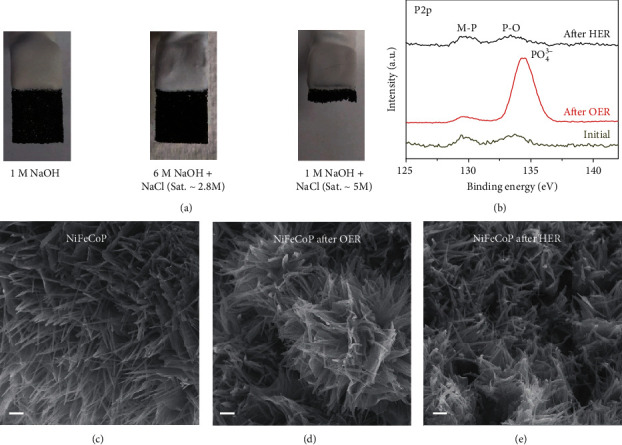
The stability of the catalysts after electrolysis. (a) Optical images of electrodes after long-term electrolysis in different electrolytes. (b) High-resolution XPS spectra of P2p obtained from NiCoFeP before and after water electrolysis. (c–e) Scanning electron microscopy images of as-prepared NiCoFeP before and after water electrolysis in 6 M NaOH + NaCl (Sat. ~2.8 M) electrolyte. The scale bar is 1 *μ*m.

Furthermore, we discovered that the proposed electrolyte was conducive to protect the electrode from corrosion and preserve the nanoarray morphology of NiCoFeP electrocatalysts, which was of extreme importance to maintain the catalytic activity and surface superaerophobicity [25]. The optical image (Figure 3(a)) illustrates that the electrode can be well preserved without structural damage even after the long-term stability test in 6 M NaOH + NaCl (Sat. ~2.8 M). On the contrary, the electrode was mostly destroyed in 1 M NaOH + NaCl (Sat. ~5.0 M) after a short time working (<2 hours). X-ray photoelectron spectroscopy (XPS, Figure 3(b) and Figure S10) demonstrated a large amount of phosphate (Figure 3(b)) [26, 27] formed on the surface of anode after long-term electrolysis in 6 M NaOH + NaCl (Sat. ~2.8 M). In the previous works, Huang and Lin [28] found that the presence of additional electrodeposited iron phosphate on the surface of CaFeO_x_ attenuates the production of corrosive hypochlorite from chloride oxidation. Hu et al. [29] found that the phosphate coating can enhance the corrosion resistance of mild steel in NaCl solution. Therefore, the phosphate formed on the surface of the anode could effectively block chloride anions [30, 31] to avoid corrosion. Meanwhile, the partial oxidation and reduction of Ni/Co in the anode and cathode, respectively, accelerate the catalytic process [26, 32–34]. Therefore, the NiCoFeP nanowire arrays on the surface of Ni foam had negligible change at the anode and cathode after the long-term stability test in 6 M NaOH + NaCl (Sat. ~2.8 M) electrolyte (SEM, Figures 3(c)–3(e)).

**Figure 4:**
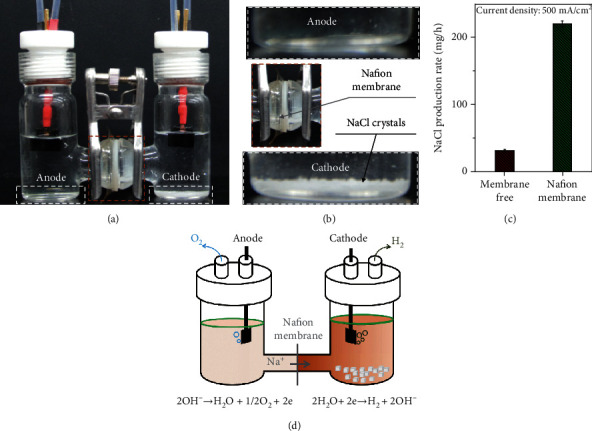
The Nafion membrane increases the yield of NaCl crystals. (a) Digital image and (b) enlarged view of the electrolyzer with membrane after long-term electrolysis. (c) The NaCl production rate in 6 M NaOH electrolyte with saturated NaCl. Error bars represent standard deviations from multiple measurements. (d) Schematic diagram of an electrolyzer for the favorable production of NaCl with the Nafion membrane.

The collection of H_2_ and O_2_ without mixing during water electrolysis is essential to achieve high-purity products [35]. The Nafion membrane was employed to separate the cathode and the anode as shown in Figure 4(a), where the membrane can facilitate the gas separation process. More interestingly, it is observed that NaCl crystals precipitated at the cathode side rather than anode side (Figures 4(a) and 4(b)). The NaCl crystal production rate in the electrolyzer with a Nafion membrane (220 mg/h) is about six times higher than that without membrane (31 mg/h, Figure 4(c)). This is because the Nafion membrane facilitates Na^+^ transfer across the membrane from the anode to the cathode during the electrolysis, leading to higher Na^+^ concentration on the cathode side, and thus speeds up crystallization of NaCl on the cathode side due to the common-ion effect. The schematic illustrations (Figure 4(d)) show the favorable production of NaCl with the Nafion membrane. The continuous supplementation of seawater to the electrolysis system during the electrolysis will gradually accumulate Na^+^ in the cathode side to trigger the crystallization of NaCl. Thus, electrolysis seawater can not only produce pure H_2_ and O_2_ but also generate NaCl crystals by inducing the super saturation state and facilitating the nucleation of crystals.

## 3. Conclusions

To generate hydrogen, a clean chemical energy, developing efficient water-electrolysis system that can work in highly corrosive, chloride-containing seawater-mimicking solution is vitally important because seawater is the most available resource, but it is a greatly challenging task. Our work shows an efficient seawater electrolysis system with triple product, namely, H_2_, O_2_, and crystalline NaCl. Due to the common-ion (Na^+^) effect in this electrolyte, the concentration of Cl^−^ can be greatly reduced, resulting in eliminated anode corrosion and ClOR, as well as NaCl crystal generation during the electrolysis process. Equipping Nafion membrane can not only effectively separate H_2_ and O_2_ but also make this electrolysis system more effective for the formation of NaCl due to the oriented Na^+^ transport capacity. The triple-production strategy demonstrated here will be a promising solution for industrial electrolysis of seawater.

## 4. Materials and Methods

The chemicals Fe (NO_3_)_3_·9H_2_O, Co (NO_3_)_2_·6H_2_O, and Ni (NO_3_)_2_·6H_2_O were purchased from Sinopharm Chemical Reagent Co, Ltd. (SCRC). NH_4_F, urea, and NaH_2_PO_2_·H_2_O were purchased from Beijing Chemical Reagents Company. Deionized water with a resistivity ≥ 18 M*Ω* was used to prepare all aqueous solutions. All the reagents were of analytical grade and were used directly without further purification. Seawater used in this manuscript was obtained from Qingdao, China.

### 4.1. Preparation of Ternary NiCoFe Hydroxide Array

The preparation of ternary NiCoFe hydroxide array followed a previous report with some modifications [16]. In a typical synthesis, 3 mmol of Co(NO_3_)_2_·6H_2_O, 1 mmol Fe(NO_3_)_3_·2H_2_O, 15 mmol of urea, and 8 mmol of NH_4_F were dissolved in 45 mL of deionized water. Then, the aqueous solution and Ni foam were transferred to Teflon-lined stainless autoclave (50 mL), sealed, and maintained at 120°C for 6 h.

### 4.2. Preparation of Ternary NiCoFe Phosphide Array

The obtained NiCoFe hydroxide array precursor and 1 g of NaH_2_PO_2_·H_2_O were placed at two positions of the porcelain boat in a tube furnace and then heated at 300°C for 2 h with a heating speed of 2°C/min in N_2_ atmosphere. After the reaction, the mass loading was about 2 mg/cm^2^.

### 4.3. Material Characterization

SEM images were obtained on a Zeiss SUPRA55 scanning electron microscope, which was operated at 20 kV. X-ray powder diffraction patterns were recorded on an X-ray diffractometer (Rigaku D/max 2500) in the range from 10° to 80° at a scan rate of 10° min^−1^. XPS was performed by using a model of ESCALAB 250.

### 4.4. Electrochemical Measurements

The electrochemical measurements for HER and OER were performed in a standard three-electrode system at room temperature on an electrochemical workstation (CHI 660D, Chenhua, Shanghai), where NiCoFeP serves as the working electrode, while carbon rod electrode and SCE electrode serve as counter electrode and reference electrode, respectively, and 6 M NaOH with saturated NaCl was used as electrolyte. In this high concentration of NaCl, the SCE was stable during the short-term electrochemical test. The CV curves were performed at a scan rate of 2 mV/s, while AC impedance measurements were conducted from 10^5^ to 1 Hz with an AC voltage of 5 mV. All potentials were converted with respect to reversible hydrogen electrode (RHE) by using the following equation: *E*_RHE_ = *E*_0_ + *E*_reference_ + 0.0592∗pH, where *E*_0_ = 0.241 V. The CV curves were *iR* corrected by using the following equation: *E*_*iR* corrected potential_ = *E*_measured potential_ − *iR* (“*R*” is the solution resistance; “*i*” is the measured current at each potential). Overall water splitting is performed in a two-electrode system in 6 M NaOH with saturated NaCl as electrolyte, where NiCoFeP are used as the cathode and anode. The gas generated from the cathode and anode during electrolysis was collected by the drainage method (measure the volume). When collecting O_2_, the gas would pass through another strong base solution to absorb the Cl_2_ that might be produced in the anode. To calculate the Faradaic efficiency, we use the following equation: FE = (*V*/*V*_m_)∗*e*∗NA∗*Z*/*Q*, where *V* is the gas volume (L), *V*_m_ is the standard molar volume (22.4 L/mol), *e* is the electron charge (1.6 × 10^−19^ C), NA is the Avogadro number (6.02 × 10^23^), *Z* is the number of electrons needed to form a molecule of gas (O_2_: *Z* = 4 and H_2_: *Z* = 2), and *Q* is the amount of electricity consumed during electrolysis (C).

## Supplementary Materials

Supplementary Materials Figure S1: schematic diagram of a single cell of an industrial electrolyzer. Figure S2: schematic diagram of the common-ion effect. Figure S3: the X-ray diffraction (XRD) patterns of formed crystals after adding 2 M KOH into saturated NaCl. Figure S4: XRD patterns of ternary NiCoFe hydroxide and phosphide (a). The red line showed the characteristic peaks of hydroxides (PDF no. 20-0786). After phosphating, all these peaks disappeared and two new peaks indexing to the characteristic peaks of Ni_2_P (PDF no. 03-0953) and Co2P (PDF no. 54-0413) appeared in the black line and blue line, suggesting complete conversion from hydroxide to phosphide on the Ni foam. Electron diffraction (ED) image (b), HAADF-STEM image (c), and corresponding energy dispersive X-ray spectroscopy (EDS) mapping images (d) of as-prepared NiCoFeP. Figure S5: CV curves of NiCoFeP on the surface of Ni foam for HER (a), OER (b), and overall water splitting (c) before and after iR compensation in 6 M NaOH electrolyte with saturated NaCl. (d–f) Chronopotentiometry curves of overall water splitting (NiCoFeP) for 100 h at different current densities before and after iR compensation using 6 M NaOH with saturated NaCl and real seawater with 6 M NaOH as the electrolyte. Figure S6: Faraday efficiency of O_2_ in different electrolytes. Figure S7: CV curves of NiCoFeP on the surface of Ni foam for overall water splitting in 6 M NaOH + saturated NaCl and real seawater +6 M NaOH. Figure S8: CV curves of OER using NiCoFeP as the catalyst in different electrolytes. Figure S9: electrochemical performance of NiCoFeP electrode. (a) Chronopotentiometry curve of overall water splitting for 112 h at 200 mA/cm^2^ without iR compensation using 6 M NaOH + NaCl (Sat. ~2.8 M) with feeding Ca/Mg-free seawater every 12 h. (b, c) CV curves of NiCoFeP for OER and HER, respectively; resistance 0.6 ohm. Figure S10: high-resolution XPS spectra of (a) Ni_2_P, (b) CO_2_P and (c) Fe_2_P obtained from NiCoFeP before and after water electrolysis. Table S1: the detailed data about the common-ion effect experiment.
