# Supplementary material for: Common-Ion Effect Triggered Highly Sustained Seawater Electrolysis with Additional NaCl Production
Source: Research (Wash D C). 2020 Sep 24;2020:2872141. doi: 10.34133/2020/2872141 (PMC7532522; doi:10.34133/2020/2872141)
Supplement: Supplementary Materials — Figure S1: schematic diagram of a single cell of an industrial electrolyzer. Figure S2: schematic diagram of the common-ion effect. Figure S3: the X-ray diffraction (XRD) patterns of formed crystals after adding 2 M KOH into saturated NaCl. Figure S4: XRD patterns of ternary NiCoFe hydroxide and phosphide (a). The red line showed the characteristic peaks of hydroxides (PDF no. 20-0786). After phosphating, all these peaks disappeared and two new peaks indexing to the characteristic peaks of Ni2P (PDF no. 03-0953) and Co2P (PDF no. 54-0413) appeared in the black line and blue line, suggesting complete conversion from hydroxide to phosphide on the Ni foam. Electron diffraction (ED) image (b), HAADF-STEM image (c), and corresponding energy dispersive X-ray spectroscopy (EDS) mapping images (d) of as-prepared NiCoFeP. Figure S5: CV curves of NiCoFeP on the surface of Ni foam for HER (a), OER (b), and overall water splitting (c) before and after iR compensation in 6 M NaOH electrolyte with saturated NaCl. (d–f) Chronopotentiometry curves of overall water splitting (NiCoFeP) for 100 h at different current densities before and after iR compensation using 6 M NaOH with saturated NaCl and real seawater with 6 M NaOH as the electrolyte. Figure S6: Faraday efficiency of O2 in different electrolytes. Figure S7: CV curves of NiCoFeP on the surface of Ni foam for overall water splitting in 6 M NaOH + saturated NaCl and real seawater +6 M NaOH. Figure S8: CV curves of OER using NiCoFeP as the catalyst in different electrolytes. Figure S9: electrochemical performance of NiCoFeP electrode. (a) Chronopotentiometry curve of overall water splitting for 112 h at 200 mA/cm2 without iR compensation using 6 M NaOH + NaCl (Sat. ~2.8 M) with feeding Ca/Mg-free seawater every 12 h. (b, c) CV curves of NiCoFeP for OER and HER, respectively; resistance 0.6 ohm. Figure S10: high-resolution XPS spectra of (a) Ni2P, (b) CO2P and (c) Fe2P obtained from NiCoFeP before and after water electrolysis. Ta [file 2872141.f1.docx]

**Supplementary Materials**

**Common-ion Effect Triggered Highly Sustained Seawater Electrolysis with Additional NaCl Production**

Pengsong Li^1, †^, Shiyuan Wang ^1, †^, Imran Ahmed Samo^1^, Xingheng Zhang^1^, Zhaolei Wang^1^, Cheng Wang^2^, Yang Li^1^, Yiyun Du^3^, Yang Zhong^1^, Congtian Cheng^1^, Wenwen Xu^4^, Xijun Liu^5^, Yun Kuang^1, *^, Zhiyi Lu^4,6, *^, Xiaoming Sun^1,^ ^*^

^1^State Key Laboratory of Chemical Resource Engineering, Beijing Advanced Innovation Centre for Soft Matter Science and Engineering, College of Chemistry, Beijing University of Chemical Technology, Beijing 100029, China

^2^Chinese Research Academy of Environmental Sciences Institution, Beijing, 100012, China

^3^State Nuclear Electric Power Planning Design & Research Institute Co., Ltd, Beijing, China

^4^Ningbo Institute of Materials Technology and Engineering, Chinese Academy of Sciences, Ningbo, 315201, Zhejiang, China

^5^ Center for Electron Microscopy and Tianjin Key Lab of Advanced Functional Porous Materials, Institute for New Energy Materials & Low-Carbon Technologies, School of Materials and Engineering, Tianjin University of Technology, Tianjin 300384, China.

^6^University of Chinese Academy of Sciences, Beijing, 100049, China

*Email: kuangyun@mail.buct.edu.cn (Yun Kuang); luzhiyi@nimte.ac.cn (Zhiyi Lu); sunxm@mail.buct.edu.cn (Xiaoming Sun)


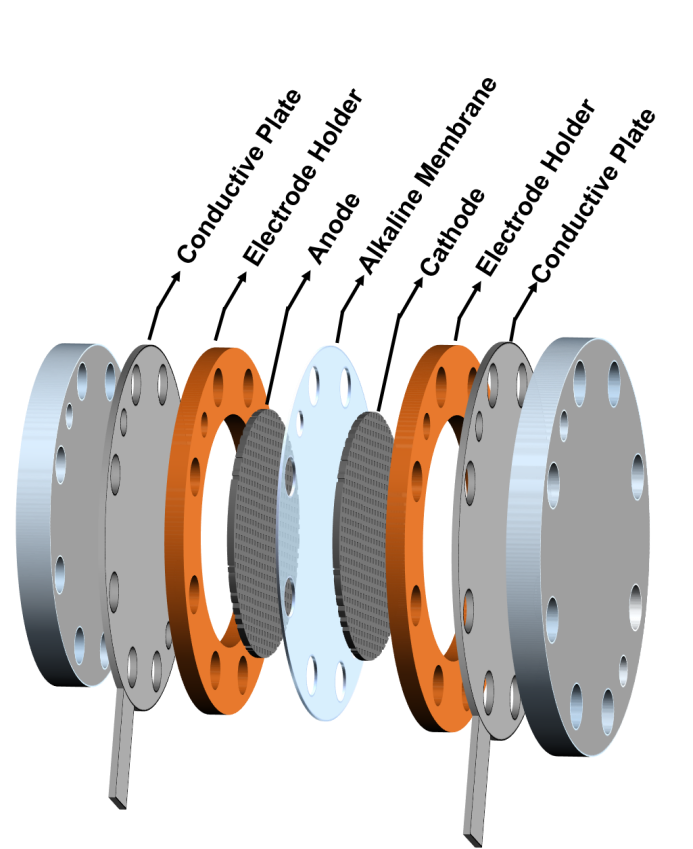


**Figure S1.** Schematic diagram of a single cell of an industrial electrolyzer.

Theoretical calculation of the concentration of NaCl during the seawater electrolysis with continuous supply of seawater:

The calculation was based on a commercial electrolyzer (electrolyzer model: HGE, Beijing CEI Technology Co. LTD). The industrial water electrolyzer is composed of several electrolytic cells. The electrode area and the working current density of the single cell is 0.196 m^2^ and 500 mA/cm^2^, respectively. One cell volume is 3 L. The initial concentration of NaCl is 0.5 M. Equation (1) is used to calculate the molar quantity of water consumption (n_H2O_), where the Faradic efficiency is assumed to be 100%. Q, N_A_ and q are total amount of charge passed through the cell, Avogadro constant and electric charge of an electron, respectively.

$n_{H2O}=\frac{Q}{2N_{A}?q}$ (1)

The theoretical concentration of NaCl(C_NaCl_) in the single cell is obtained from Equation (2), where V_as_ is the volume of seawater feeding per hour,. V_cell_ is volume of single cell and t is the running time of single cell.

$C_{NaCl}=0.5+\frac{0.5V_{as}}{V_{cell}}?t$ (2)

In the system of pure seawater as electrolyte without adding NaOH, the initial and saturated concentration of NaCl is 0.5 M and 5.33 M, respectively. With feeding seawater per hour, NaCl crystals will precipitate from electrolyte after 88 h electrolysis. In the electrolyte of 6 M NaOH and 0.5 M NaCl, the saturated concentration of NaCl is 2.8 M. With feeding seawater per hour, NaCl crystals will precipitate from electrolyte after 42 h electrolysis.


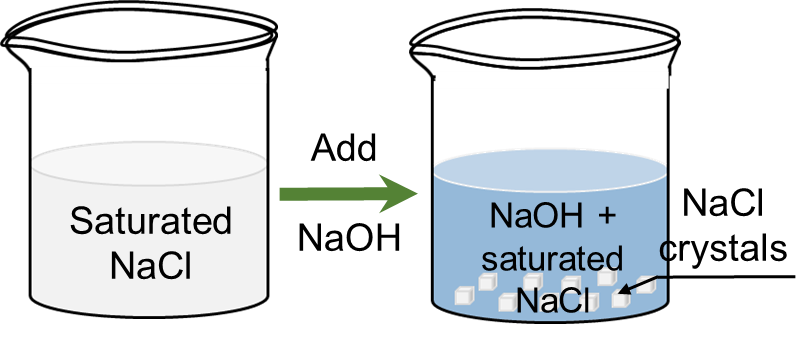


**Figure S2.** Schematic diagram of common-ion effect.


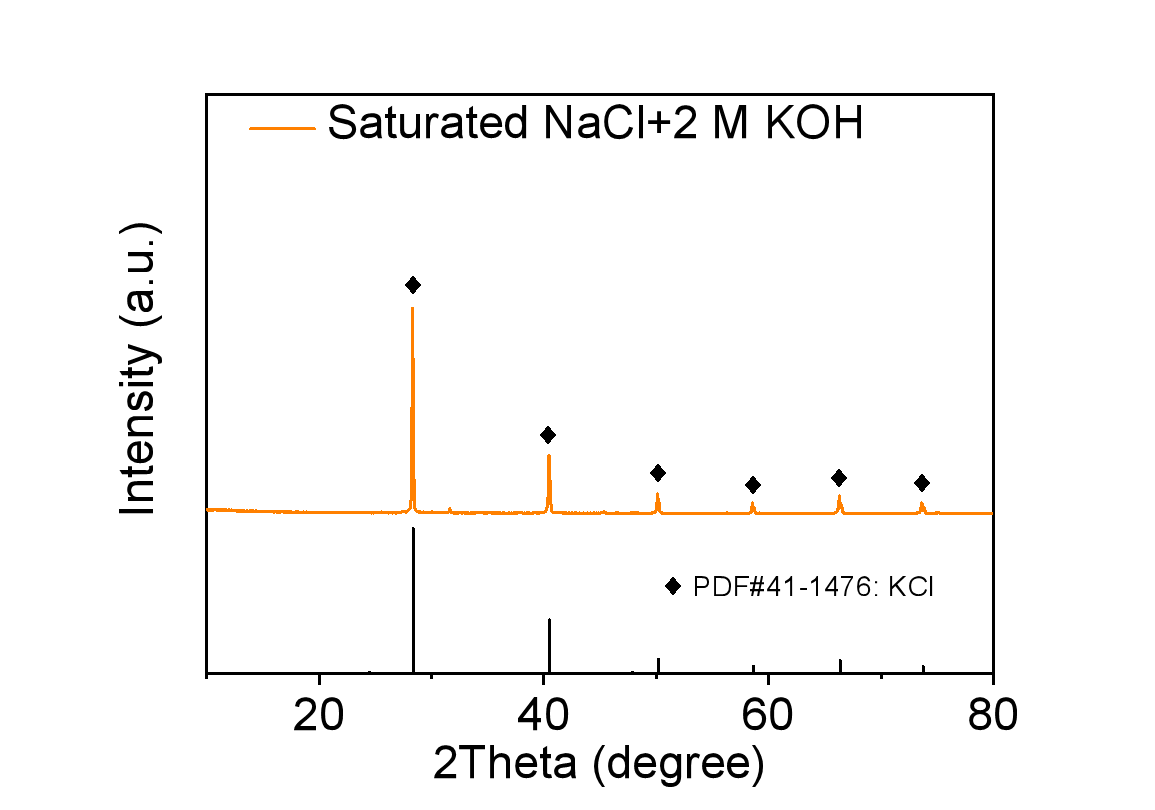


**Figure S3.** The X-ray diffraction (XRD) patterns of formed crystals after adding 2 M KOH into saturated NaCl.


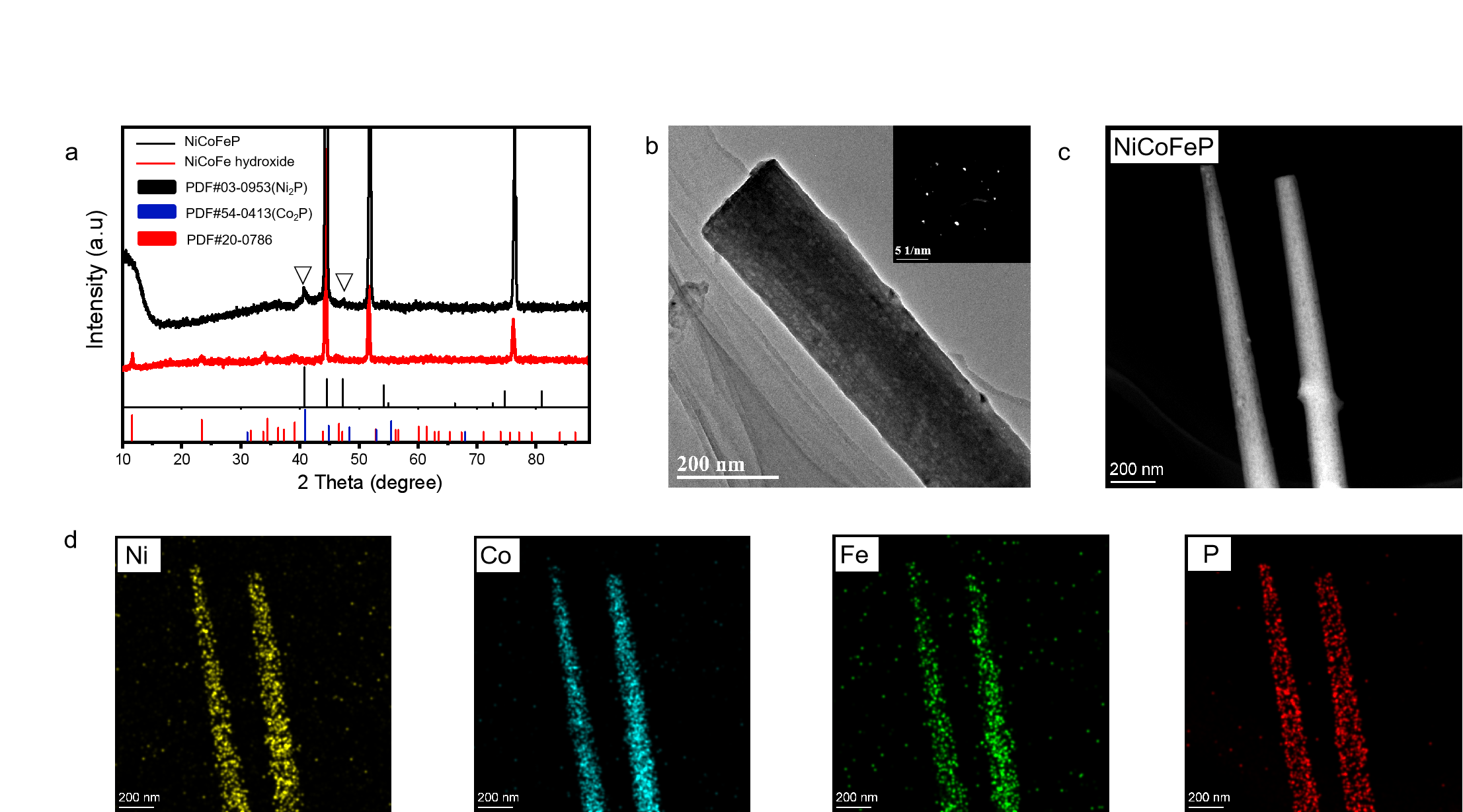


**Figure S4.** XRD patterns of ternary NiCoFe hydroxide and phosphide (a). The red line showed the characteristic peaks of hydroxides (PDF#20-0786). After phosphating, all these peaks disappeared and two new peaks indexing to the characteristic peaks of Ni_2_P (PDF#03-0953) and Co_2_P (PDF#54-0413) appeared in the black line and blue line, suggesting complete conversion from hydroxide to phosphide on the Ni foam. Electron diffraction (ED) image (b), HAADF-STEM image (c) and corresponding energy dispersive X-ray spectroscopy (EDS) mapping images (d) of as-prepared NiCoFeP.

**
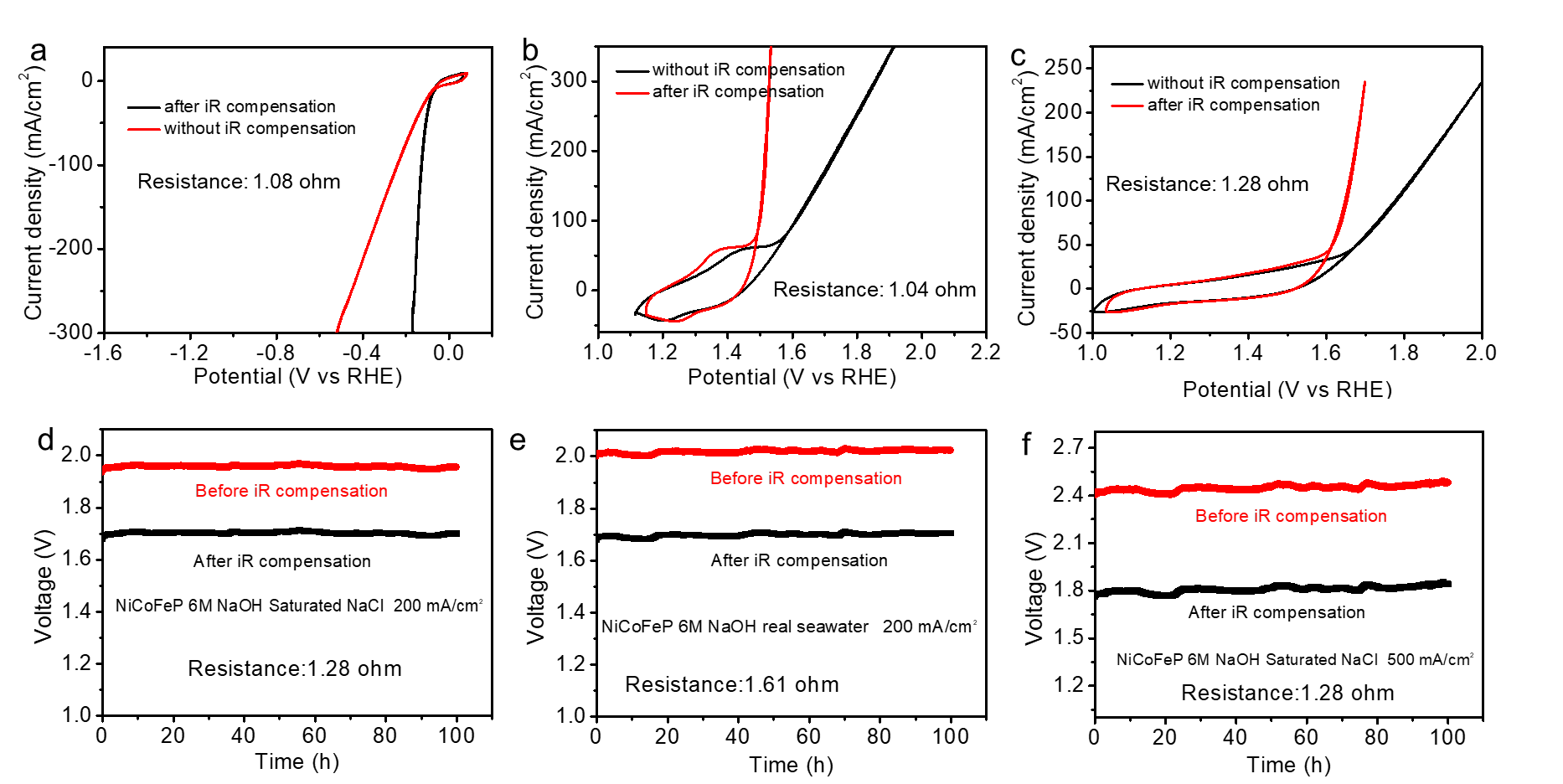
**

**Figure S5.** CV curves of NiCoFeP on the surface of Ni foam for HER (a), OER (b) and overall water splitting (c) before and after iR compensation in 6 M NaOH electrolyte with saturated NaCl. (d, e, f) Chronopotentiometry curves of overall water splitting (NiCoFeP) for 100 h at different current densities before and after iR compensation using 6 M NaOH with saturated NaCl and real seawater with 6 M NaOH as the electrolyte.


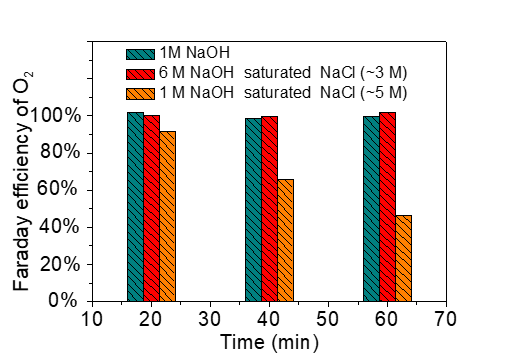


**Figure S6.** Faraday efficiency of O_2_ in different electrolytes.


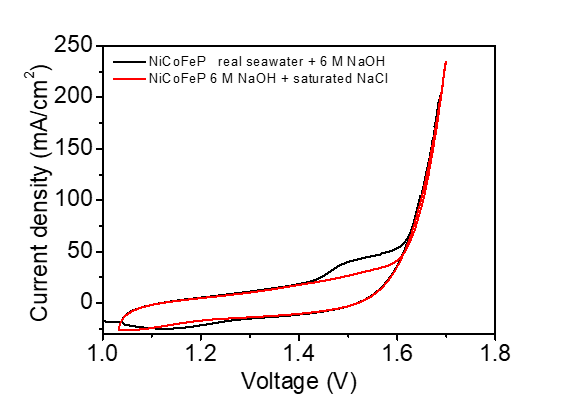


**Figure S7.** CV curves of NiCoFeP on the surface of Ni foam for overall water splitting in 6 M NaOH + saturated NaCl and real seawater + 6 M NaOH.


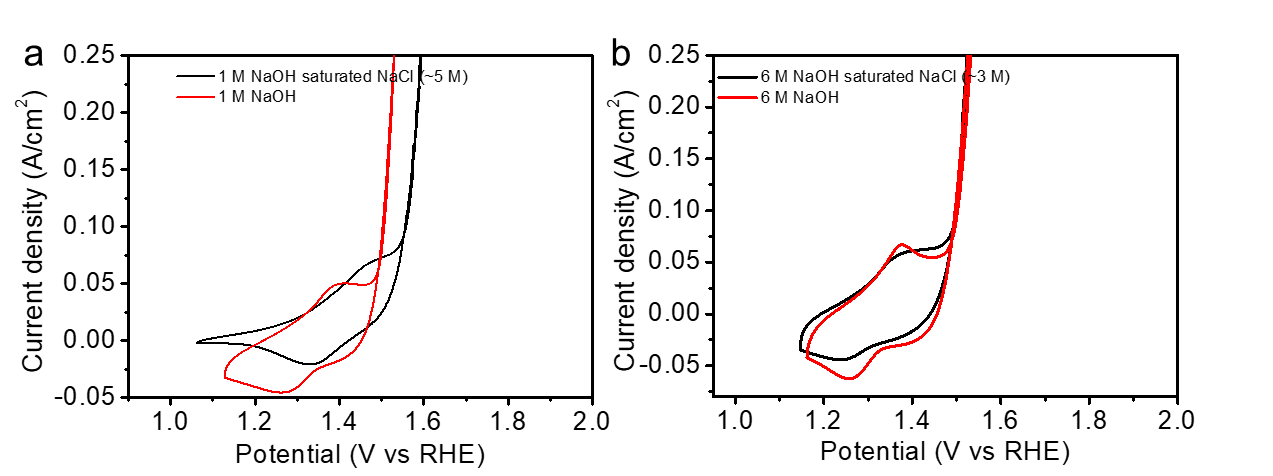


**Figure S8.** CV curves of OER using NiCoFeP as the catalyst in different electrolytes.


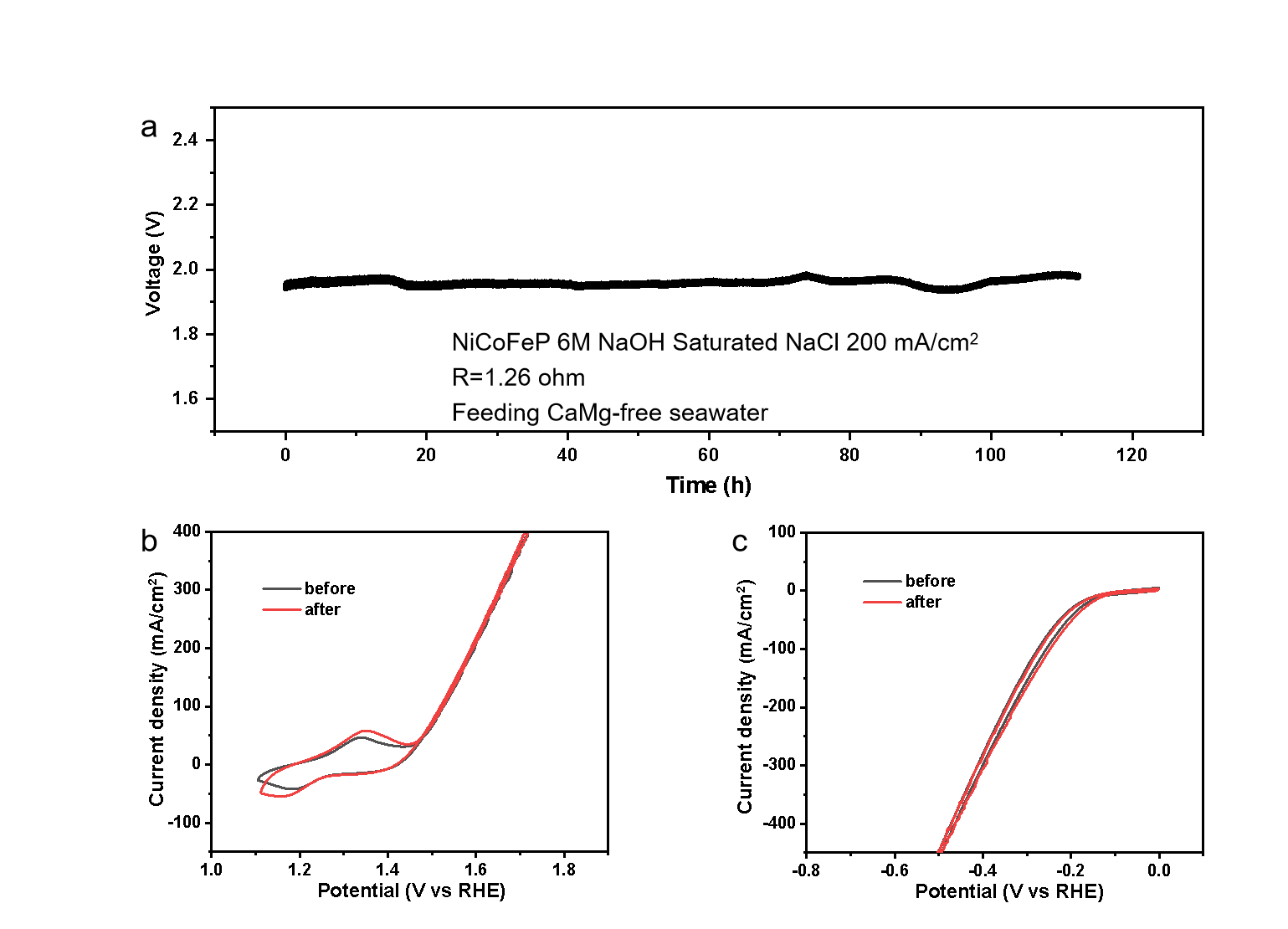


**Figure S9** Electrochemical performance of NiCoFeP electrode. (a) Chronopotentiometry curve of overall water splitting for 112 h at 200 mA/cm^2^ without iR compensation using 6 M NaOH + NaCl (Sat. ~2.8 M) with feeding CaMg-free seawater every 12 h. (b, c) CV curves of NiCoFeP for OER and HER respectively, resistance 0.6 ohm.


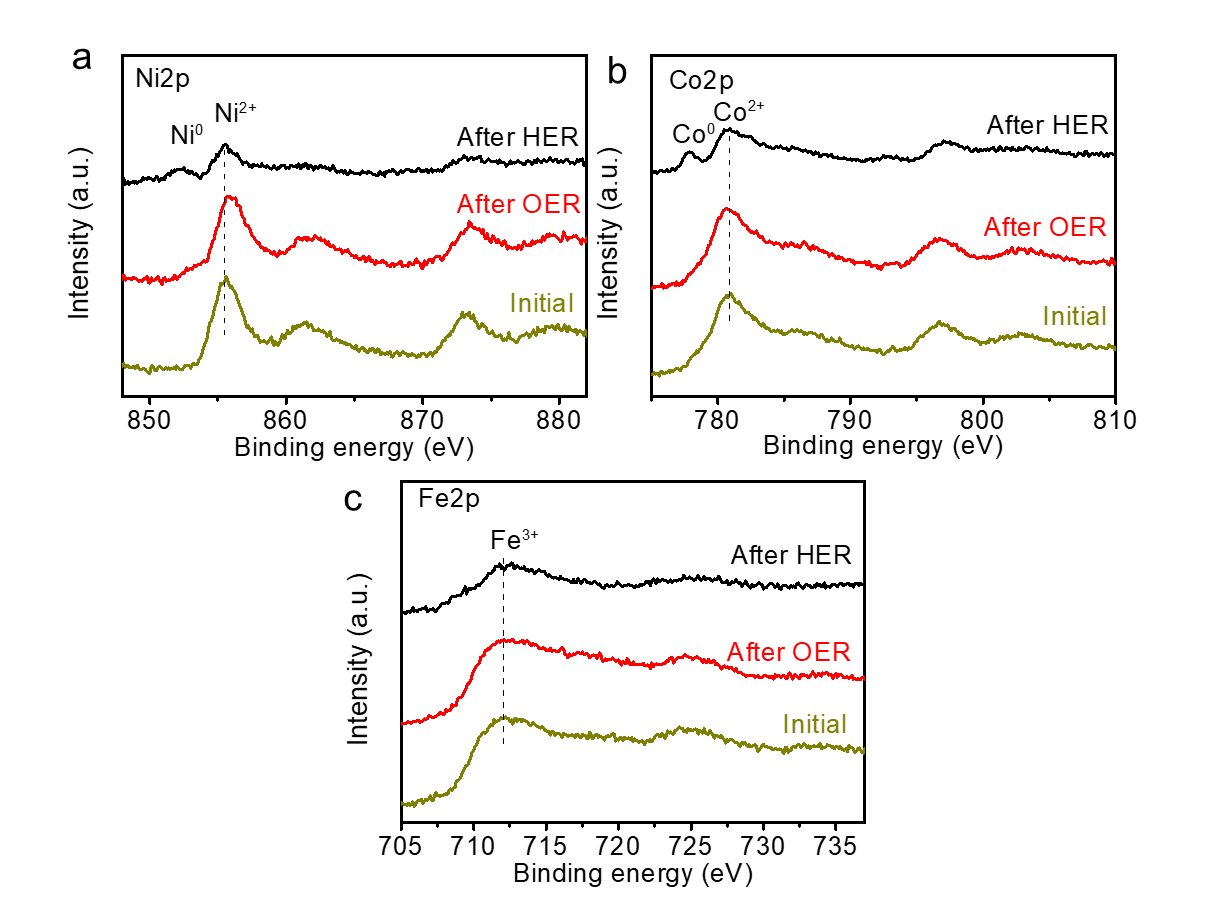


**Figure S10.** High-resolution XPS spectra of (a) Ni 2p, (b) Co 2p and (c) Fe 2p obtained from NiCoFeP before and after water electrolysis. The Ni and Co in the anode were oxidized to higher oxidation state to accelerate OER, as evidenced by the red shift (Ni 2p) and the blue shift (Co 2p), respectively. The peaks of metallic state (Ni and Co) emerged in the cathode, indicating the metal elements were reduced during the electrolysis process. The chemical state of Fe was retained after OER and HER.

**Table S1**. The detailed data about the common-ion effect experiment

| Number | 1 | 2 | 3 | 4 | 5 |
| --- | --- | --- | --- | --- | --- |
| NaCl (g) | 35.2 | 3.4 | 4.1 | 5.9 | 4.5 |
| Volume of solution (mL) | 113 | 110 | 108 | 105 | 103 |
| NaOH (mg) | 0 | 4 | 8 | 16 | 24 |

To measure the solubility of NaCl at different concentrations of NaOH, we performed the following procedure. Firstly, we added 35.2 g NaCl into 100 g water to prepare the saturated solution (marked as solution 1), and the volume of solution increased from 100 mL to 113 mL. Secondly, we added 4 g NaOH into above saturated NaCl solution (solution 1) with string for 6 h, then filtrating the solution (marked as solution 2) and the sediment (NaCl). The volume of this solution 2 was 110 mL containing 4 g NaOH. The sediment (NaCl) was 3.4 g after drying. Thirdly, we added another 4 g NaOH into above saturated NaCl solution with string for 6 h, then filtrating the solution (marked as solution 3) and the sediment (NaCl). The volume of this solution 3 was 108 mL containing 8 g NaOH. The sediment (NaCl) was 4.1 g after drying. Fourthly, we added another 8 g NaOH into above saturated NaCl solution with string for 6 h, then filtrating the solution (marked as number 4) and the sediment (NaCl). The volume of this solution 4 was 105 mL containing 16 g NaOH. The sediment (NaCl) was 5.9 g after drying. Finally, we added another 8 g NaOH into above saturated NaCl solution with string for 6 h, then filtrating the solution (marked as number 5) and the sediment (NaCl). The volume of this solution 5 was 103 mL containing 24 g NaOH. The sediment (NaCl) was 4.5 g after drying.
